# The Restoration of Badly Broken Teeth without Crowning

**Published:** 1897-04

**Authors:** S. E. Davenport

**Affiliations:** New York


### THE RESTORATION OF BADLY BROKEN TEETH WITHOUT CROWNING.[1]

BY S. E. DAVENPORT, D.D.S., M.D.S., NEW YORK.

The above title is intended to express the writer's preference for built-up, patched, banded, and riveted natural teeth over the same teeth cut off to or near the gum and crowned.

The dental profession, itself a specialty, is being of late subdivided into specialties, of which the most popular one, "crown- and bridge-work," is by some unscrupulous men practised because of its advertising and money-making qualities, and even in the hands of conscientious men is often ridden as a hobby far beyond its value.

---

[1] Read before the Massachusetts Dental Society at Boston, June 3, 1896.

The writer desires to place himself on record as in favor of both crowns and bridges whenever and wherever the conditions of the mouth and remaining teeth (not the patient's pocket) justify their use; but he desires to emphatically condemn the sacrifice of either whole teeth or parts of teeth for the purpose of crowning or bridging where a more conservative plan is available.

It is hoped that this little paper will direct attention to what the writer believes to be a serious fault in the practice of many dentists, due to the great improvement in the methods of crowning and bridging and to the mass of information concerning the same, which is being spread broadcast in the journals.

The impression made upon the mind of the average dentist by all of this is so great that when he is consulted concerning a broken tooth he immediately considers the various methods of crowning, and decides to use one of them, while, if he would give the same amount of thought and ingenuity to the devising of a plan for the preservation of what remains of the tooth and a restoration of the broken portions with some strong material, he would not only give his patient in the majority of cases a more useful organ, but he would not be taking the very last possible step for the preservation of that tooth.

Crowning should be, just as it is, in fact, *the last resort!*

Many instances illustrating the abuse of crowning could be cited from the writer's observation, but he will confine himself to a description of a few general classes of cases.

Occasionally he has removed gold crowns for the purpose of relieving painful conditions, and has found underneath live teeth so good, even after the wholesale grinding to which they had been subjected in the fitting of the crown, that he has been able to restore their contour and usefulness with fillings.

Both gold and porcelain crowns have been seen by the writer in the mouths of patients under fourteen years of age, children of well-to-do parents, the other teeth being of average strength.

Such cases are always from the hands of some practitioner who "makes a specialty" of crowning. Dentists of this stamp do not hesitate to condemn molars and bicuspids having ordinary compound approximal cavities to a hollow gold crown, positive assertion being made to the patient that the tooth is beyond hope of safety from fillings.

Such crowns are usually put on without regard to contour, and as a consequence food crowds in and decay begins at the gum. Of course, when a tooth is once cut off and crowned it is almost impossible for other dentists to decide whether crowning was necessary or not, as all direct evidence is usually destroyed. The general condition of the other teeth and the density of the tooth-substance are about the only guides remaining, unless some opinion can be formed from a knowledge of the dentist whose judgment was followed.

One very weak point in crowning is the almost universal use of zinc phosphate for the cementing material. Gutta-percha is so much more reliable and durable as a cement, besides allowing the crown to be removed upon the application of heat whenever necessary, that it seems strange more operators do not use it in preference to the phosphate cements.

Amalgam properly selected, prepared, and packed is, in the large majority of mouths, a very durable material, and it is, of course, the sheet-anchor in the building up of broken teeth, with the possible exception of the incisors, even though porcelain or gold, or a combination of the two, may be used for the purpose of hiding the amalgam from view.

Dr. George S. Allan, of New York, in his recent able paper upon the subject of contouring, justly places amalgam among the "constants" of his equation, and whenever a method of restoration or partial crowning can be adopted which will admit of the use of amalgam in place of the usual zinc phosphate, a great element of strength and durability is certainly added to the finished operation.

While the writer would prefer to leave to every dentist's ingenuity the methods most suitable for the restoration of broken teeth, provided each operator would give his thought to the preservation of what remains, rather than to the cutting away of everything in sight preparatory to crowning, it may not be out of place to briefly describe a few methods which have been of great service to the writer, and may, when modified to suit the special case in hand, be of some help to others.

Before describing any certain methods it will, perhaps, be well to say a few words about some of the mechanical devices common to many operations. Metal pins for use in the roots of pulpless teeth as anchorage posts may be made of platinum alloyed with iridium, gold alloyed with platinum, or of stiff German silver wire, the latter being, of course, much less expensive but not quite as reliable for the demands in all respects as the two first named.

This wire in several different sizes (say Nos. 3 to 7 of bur-gauge) should always be at hand, and before being used a shallow screw-thread should be cut upon it merely for the purpose of giving proper hold to whatever plastic material may be used in connection with it.

The writer prefers to select a screw a trifle smaller than the calibre of the prepared root-canal, and cement it in place with zinc phosphate, rather than to use a screw slightly larger than the canal, depending upon the thread cut in the dentine by screwing it in, as some operators do. What has been said above against the use of zinc phosphate refers, of course, only to its use in locations reached by the saliva. Stiff German silver is not as rigid as the other wires referred to, but will always answer if two roots can be utilized, a pin being placed in each. It is not affected by mercury, nor is ordinary clasp metal—gold and platinum—by the proportion of mercury in amalgam, though pure mercury will sometimes affect it slightly. Stiff German silver has been referred to because there are two varieties of the metal obtainable. The largest round bur used in preparing the root-canal for the reception of the metal pin should be measured in the bur-gauge, and a pin just one size smaller selected for use.

The next adjunct of importance in many of these operations is the ring matrix of thin steel or German silver, which every dentist should have at hand in all sizes and widths.

They are easily made, a little muriate of zinc and soft solder only being necessary to join the ends, care being taken to place the point of union against tooth-structure when they are to be used with amalgam, so that the solder may not be eaten by the mercury. Thin steel of a variety of thicknesses suitable for the making of such matrices is now obtainable at most dental depots, though at a higher price, as is usually the case, than if the same be purchased of houses in that special line of business. They are useful daily to assist in the shaping of plastic fillings, and can often be made to serve as a help in contouring with gold or gold and tin. Their use is also indicated to encircle teeth having approximal decay extending some distance below the gum, the rubber dam being easily applied after the placing of the matrix.

A box, something like the one now shown with numbered pins for holding and separating the ring matrices according to size, is easily constructed, and with the measuring mandrel completes a most useful adjunct to any office.

Gold-faced platinum, which is pure gold welded upon pure platinum, is so useful in many cases of reconstruction that it should be referred to here with the other standard helps. It is very soft and can easily be made to conform to any surface, and the platinum side is, of course, valuable when amalgam is to be used.

For ordinary permanent bands of gold-faced platinum, No. 32 is about the right thickness, though when a split molar is to be banded a heavier number would be better, because of the great strain which would come upon it.

One of the most common accidents which dentists are called upon to repair is the superior bicuspid from which either the buccal or palatal face has broken, often to the gum line, carrying away frequently both approximal fillings. Such teeth are usually pulpless, and the remaining portions of the crown can be retained, and the contour restored either with amalgam or with a combination of amalgam and porcelain as follows:

If the buccal wall is still standing, a strong metal pin should be cemented into the root and left nearly as long as the finished palatal portion is designed to be. All soft and frail edges having been cut away, a temporary band matrix, similar to those shown, is selected of a size suitable to represent the proper contour of the finished tooth, and is put in place outside of and around the broken crown and the pin, care being taken to fit the matrix accurately to the gum line above the break.

The ring matrix is then filled carefully and solidly with amalgam, the first pieces quite plastic, to enter all inequalities about the pin and broken tooth, but hard and dry amalgam should be used when the operation nears completion. The ring should be left on the tooth for at least six hours to support the amalgam until fully hard, when it should be removed and the amalgam shaped and polished.

Should the buccal wall instead of the palatine be broken off, a cross pin porcelain facing of the proper size and color should be selected and ground to fit the buccal aspect of the tooth just under the edge of the gum. The pins of the porcelain are now fitted into a narrow scrap of platinum, or gold and platinum, and soldered with eighteen carat solder. This cross-piece of metal is attached to the pins near their ends at a sufficient distance from the facing to allow a screw to pass through the square opening thus made. The screw is then cemented into the root, the porcelain placed in its proper position with its joined pins encircling the screw, and a temporary band matrix of proper size is used to surround the various parts of this combination.

Enumerating these parts from without inward, there are the steel band, the porcelain facing, the screw, the palatal wall of the natural tooth, and again the steel matrix. Amalgam is then packed into the matrix and all about the pins of the porcelain, the screw which is secured far up in the root, and into the inequalities of the palatal wall of the tooth.

When the matrix is removed the next day and the amalgam finished, the result is most pleasing. This method of repairing pulpless bicuspids from which the buccal wall has broken was ably described by Dr. E. A. Bogue, of New York, in the *Dental Cosmos* about ten years ago.

Should the broken bicuspid have a living pulp the palatal portion could be contoured with amalgam packed into a band matrix, a good anchorage being obtained at the cervical wall, and care being taken not to build the filling out to the shape of a normal cusp.

When the buccal wall of a living bicuspid breaks, the porcelain selected should be backed with a piece of thin gold-faced platinum, the backing being extended to form a permanent band around the palatal wall of the tooth and amalgam being built into the band between the porcelain and the remaining tooth wall.

Should it be a second bicuspid in a man's mouth, a porcelain facing would not always be necessary, in which case a permanent band of gold-faced platinum should be carefully fitted around the remaining wall and the neck of the tooth and filled in solidly with amalgam. This band should be almost as deep as the required length of the tooth.

Where molars break so badly as to prevent the use of any of the above-described methods, the writer prefers often to use an open band of gold-faced platinum fitted carefully to the gum line of the remaining portions of the tooth rather than a finished and more æsthetic gold crown, for the reason that after cementing the screws into the roots and placing the fitted band in position a good view of the interior can be obtained while amalgam is being packed against the broken margins of the tooth and about the screws until the band is full.

Strict contour can be practised in all these methods, care being used in the selection of the ring matrices and in the fitting of the permanent bands, the material of which both are constructed being so thin as to be easily burnished out to even an exaggerated contour before the introduction of the amalgam.

When two or more screws can be anchored well down in molar roots it is perfectly safe, and adds to the good appearance of the finished operation to groove the amalgam at a subsequent sitting and cover it with gold.

Aside from the improved appearance, gold certainly makes a better and safer masticating surface than amalgam almost anywhere, the amalgam being so brittle that small pieces chip off from those margins which receive the greatest force of occlusion, decay resulting.

So hard is amalgam that it does not wear down to keep pace with the surrounding tooth-substance, which fact causes it often to act as a wedge, thus helping to destroy the tooth it was intended to preserve.

Devitalized superior cuspids often break seriously, leaving but a shell which would not of itself hold any sort of filling for a great length of time. It seems to be the general custom to crown such teeth, but should there be enough of the labial enamel remaining to give a fairly good appearance, the writer derives more satisfaction and he believes a stronger result from cementing a screw into the root and restoring the contour with amalgam, zinc phosphate being used against the thin labial enamel to prevent the amalgam from showing through. At the next sitting the amalgam in sight is grooved and covered with gold.

A word should be said in commendation of those conservative operators who have withstood the onward march of incisor crowns of all descriptions, their great manipulative skill and knowledge of the principles of dental architecture enabling them to restore with gold the portions lost by decay, accident, or wear, often preserving pulps alive and postponing for perhaps ten years or more the evil day when a crown may become necessary.

Much pleasure is also taken in referring here to the "gold bandage" devised by Dr. J. F. Adams, of Worcester, a description of which was published in the *Dental Digest* for January, 1896.

It is certainly a most valuable expedient for preventing fracture and decay of frail teeth and for the protection of worn and eroded surfaces.

The idea is susceptible of considerable expansion, and it is probable that all who take advantage of Dr. Adams's originality will find extended and varied uses for the gold bandage.

The writer has presented a few methods of more or less value for which he makes no claim of originality, being something of a coward, and having had a little experience in dental societies where such claims were being discussed; he therefore asks his hearers to adopt freely whatever may seem of value in the methods described, without fear of encroaching upon the proprietary rights of any one.

It is probable that a better collection of methods for restoring broken teeth could be offered by many present, but as the writer has taken upon himself the privilege of urging his hearers to give first thoughts to preservation rather than to destruction and crowning, it seemed necessary, in order to make his position tenable, to offer certain suggestions in the light of personal experience.

While it would be possible to extend the above list and describe in detail many peculiar operations, emergency cases from practice, such as the banding of split molars, the riveting back into position portions of enamel separated from a tooth by accident, etc., the writer has chosen to refer only to such general classes of cases as are frequently met with, feeling sure that if his hearers will adopt the methods described, with such modifications as ingenuity suggests and the special case makes necessary, so many advantages over ordinary crowns will be observed that each operator will of his own volition improve and extend the system until his creed will read "*preserve and reinforce*" rather than as it seems to in these latter days "*destroy and crown!*"

It is to be hoped that all dentists will bear in mind the high character of their calling,—the preservation of teeth!

